# Patient’s views of the consent process for groin hernia repair: Use of consent template improves compliance with best practice (Original research)

**DOI:** 10.1016/j.amsu.2018.09.033

**Published:** 2018-09-25

**Authors:** Saad U. Khan, David J. Bowrey, Robert N. Williams, Jun Yi Soh, Aikaterini Peleki, Nazli Muhibullah, Peter W. Waterland

**Affiliations:** a Russells Hall Hospital, University Hospitals of Leicester, 18 Glebe Road, Leicester, LE6 0GT, UK; b University Hospitals of Leicester NHS Trust, Leicester Royal Infirmary, LE1 5WW, UK; c University of Birmingham, Russell's Hall Hospital, Pensnett Rd, Dudley, DY1 2HQ, UK; d Russells Hall Hospital, Pensnett Rd, Dudley, DY1 2HQ, UK; e Nottingham City Hospital, Hucknall Rd, Nottingham, NG5 1PB, UK

**Keywords:** Inguinal hernia, Hernia, Herniorrhaphy, Informed consent, Surveys and questionnaires, Patient satisfaction

## Abstract

**Background:**

Informed consent obtained for day case surgery has been historically incomplete. An assessment of consenting practice for groin hernia was performed relative to existing gold standards and patient's perception of the consent process was evaluated with a questionnaire. The aim of the study was to identify areas of improvement to comply with best practice.

**Methods:**

A retrospective audit of adult patients undergoing groin hernia repair (June–November 2016) at a tertiary care centre was performed. The same cohort of patients was surveyed with a self-administered questionnaire to identify their view on consenting practice.

**Results:**

113 patients were identified who underwent groin hernia repair during the study period. Pre-printed consent templates-stickers (as opposed to hand-written) were used in 53(47%) cases. In 75(66%) cases, there was complete documentation of the risks and benefits of surgery. 81(72%) patients received information about the full benefits of surgery. 27(23%) patients received partial information and 7(6%) patients had no mention of benefit recorded. Postoperative recovery was fully explained to 85(75%) patients. Use of pre-printed templates ensured 100% documentation compared to handwritten consent forms (risks 37%, benefits 47%, and recovery 53%). Preference for the timing of consent was in clinic (64%), day of surgery (25%). 34(56%) felt the choice for the technique and 22(36%) felt the choice for anaesthesia. Satisfaction was non-significantly better in those consented in clinic (87% versus 76% p = 0.74). 49(80%) felt happy with the overall consent process. 57(93%) felt that they received support and advice. 60(98%) responders felt confidence in the National Health Service and 59(97%) would recommend treatment to family and friends.

**Conclusions:**

The use of pre-printed consent and discharge summary templates improve compliance with best practice. Whilst patient preference favours consent in the outpatient clinic, satisfaction levels were high wherever consent was obtained. Patients should have more choice.

## Highlights

• Informed consent is the legal and ethical right of every patient who is able to give consent.
• Informed consent obtained for day case surgery has been historically incomplete.
• There is still a degree of medical paternalism.
• Significant number of the patients prefer consent in the clinic in advance of the day of surgery.
• Use of pre-printed consent and discharge summary templates improve compliance with best practice.

## 1 Introduction

Groin hernia repair is the most common general surgical operation performed, with over 71,000 procedures undertaken each year in England [1]. A person-centered approach is central to delivering high quality care in the modern National Health Service (NHS) [2]. Informed consent is the basic legal and ethical right of all patients able to make decisions about their healthcare and treatment, it is based on the fundamental principle of autonomy; one of the four pillars of medical ethics (autonomy, beneficence, non-maleficence and justice) [3,4].

The consent process should be an uncoerced and voluntary decision of a competent person based upon adequate information [5]. This begins at the first consultation with a discussion between healthcare professional and patient about the nature, benefits, risk and alternatives of the proposed treatment. Every detail of the consent process must be documented carefully. Patient information leaflets form an important tool in this regard. The healthcare professional undertaking the procedure is responsible for obtaining the consent. Where this is not practicable then this may be delegated to a person who is suitably trained, has sufficient knowledge of the proposed investigation or treatment, and understands the risks involved. A delegated consent needs to be confirmed by the responsible clinician before the start of the investigation or treatment.

An undisclosed risk may potentially give rise to unrealistic expectations, patient dissatisfaction, negligence claims and in some cases, criminal charges [6]. These can cause significant financial impact on the healthcare system. Following a Supreme Court judgment in the case “Montgomery v Lanarkshire Health Board” (2015), the law now requires the doctors to disclose any material risks involved in a proposed treatment and reasonable alternatives [7].

We performed a retrospective study of consenting practice prior to groin hernia repair and surveyed the same patient cohort with a self-administered postal questionnaire. The aim of this study was to evaluate whether use of a standardized template improves compliance with national standards compared to when not used. Also to assess patient perception of the consent process, including the optimal timing for obtaining consent prior to surgery.

## 2 Methods

A retrospective audit of adult patients undergoing elective groin hernia operation over a six month period (between 1 June and 30 November 2016) at the University Hospitals of Leicester was performed. Institutional approval from the clinical audit standards and effectiveness board was obtained prior to commencement. British hernia society and European Hernia society criteria were selected as a gold standard [[8], [9], [10]] (Table 2). Patients undergoing emergency hernia repair or aged less than 18 years old were excluded. Patients were identified using the Operating Room Management Information System (ORMIS) and the medical records of patients were reviewed retrospectively to obtain the following parameters: patient demographics, outpatient consultation letters, consent form data, operation notes, hospital discharge letters, and grade of health care professional involvement, record of significant and frequent complications. The quality of the data was dependent on the documentation in the clinical notes. After confirmation of diagnosis, the consent process starts during the initial consultation, treatment options pros and cons of the proposed treatment are explained. The patient is provided with a patient-friendly information leaflet around the time he/she is booked, or pre-assessed for surgery. The signing of written consent form is variable either at the time of clinic or day of surgery.

**Table 1:** Patient survey questionnaire.

Domain	Questions and response
Place or timing of written consent	Where were you asked to sign the consent form?
	At the first clinic appointment
	At the second clinic appointment or On the day of operation
Your opinion on the best time for the consent.	At the first appointment
	To be given information first and obtain at the next appointment
	On the day of operation
	Any of the above, and other comments
The amount of Information provided, and time allowed	Do you think you had enough time to make your decision? (Y/N)
	Was procedure adequately explained the way you could understand & did you feel you received enough information to make decision? yes fully, to some extent, no,
	Were the benefits, risks & possible alternatives explained? (Yes fully, to some extent, no)
	Could you change your mind or withdraw consent? (Y/N)
	Were you given information about the anaesthetic technique? yes fully, to some extent, no
	Did you receive written information or leaflet? (Y/N)
Choice	Did you have a choice in the procedure (e.g. Open operation, key hole)? Y/N
	Did you have a choice with regards to anaesthetic technique (e.g. done while asleep or make it numb while awake)?
Postoperative care	Were you informed about normal activities e.g. self-care, driving, light work, return to work, operating machinery, signing legal documents, drinking alcohol? (yes fully, to some extent, no)
	Did you experience pain after operation? (Y/N)
	How was it controlled (pain killers from the hospital, saw GP, re-admitted)?
	Did the operation improve your symptoms? (Y/N)
	Did you receive effective treatment, advice and support? (Y/N)
Waiting times	Approximately how long did you have to wait to see surgeon? (<1 month, 1–4 months, >4 months)
	Was this reasonable? (Y/N)
	How long did you have to wait in the clinic? (<30 min, >30 min)?
	Was this reasonable? (Y/N)
	How long did you have to wait for operation after decision was made? (<4 moths, >4 months)?
	Was this reasonable? (Y/N)
Overall Opinion	Did you have pain after the operation? (Y/N)
	How was this managed? (self-medication, GP, re-admission)
	Did you have confidence and trust in the health care person who was treating/advising you? (Yes/no)?
	Would you recommend the service to your family and friends? (yes/no)

**Table 2:** Best practice guidance and audit results: Hand written versus consent templates.

Best practice guidance	Consent template used (n = 53)	Hand written consent (n = 60)	P-value	Total
**Benefits of the procedure**	53 (100%)	28 (47%)	0.0001	81 (72%)
•Reason for and nature of the procedure				
•Relief of symptoms				
•Prevent complications)				
**Choice of LA**	2 (4%)	5 (18%)	0.045	7 (6%)
• Elderly, significant co-morbidities or any patient undergoing open procedure.				
**Significant complications associated with groin hernia repair**	53 (100%)	22 (37%)	0.0001	75 (66%)
• Self-limited neuralgia: 10-20%				
• Chronic pain: 10-12%				
• Haematoma: 5-16%				
• Seroma: 1-12%				
• Recurrence: 1-5%/5Y				
• Urinary retention: LA 0.37%, GA 2-3%				
• Wound infection:1-3% open, 0.32-0.65% Lap				
• Testicular complication: 0.5-1%				
• Bladder damage: uncommon, open/lap, 0.2%				
• Vas Injury: 0.3-2%				
• Mortality: (same as general population) 0-0.02% ^11^				
• Mesh infection: < 0.5%	0 (0%)	4 (16%)	0.0001	4 (4%)
• Numbness: Less after lap	0 (0%)	4 (16%)	0.0001	4 (4%)
**Complications specific to laparoscopic repair**	n = 13	n = 29		
Intestinal obstruction (TAPP): 0.07-0.4%	0 (0%)	0 (0%)	1	0 (0%)
Port site hernia: 1%	0 (0%)	0 (0%)	1	0 (0%)
Intestinal/visceral injury: 0-0.21%	13 (100%)	21 (72%)	0.0427	34 (30%)
Vascular injury: 0.06-0.13	13(100%)	20 (69%)	0.0382	33 (29%)
**Discharge summary**	53 (100%)	32 (53%)	0.0001	75 (66%)
Pain control, return to routine activity, driving, work, red flag signs and symptoms.				

In the second part of the study, the same cohort of patients was sent a self-administered, 6 dimensions, and 31 item questionnaire by postal mail (Table 1).

The statistical software package Statistical Package for the Social Sciences 20 (SPSS 20) was used to perform statistical analysis. The median was used as a measure of the central tendency for continuous variables. Pearson's chi-square test was employed for comparison of categorical variables. A p value of <0.05 (2-tailed) was deemed statistically significant.

## 3 Results

The study population was 115 patients undergoing elective groin hernia repair during the time period 1 June to 30 November 2016. In two cases the written consent forms were missing from the medical notes but other information was available, including operation notes, clinic letter and discharge summary, these were excluded from the study.

109 (96%) were males, and 4 (4%) females. Only one (1%) patient underwent femoral hernia repair, 93 (81%) patients had primary unilateral hernia repair, 6 (5%) primary bilateral inguinal repair, 3 (3%) recurrent inguinal repair and 13 (11%) had previous contralateral surgery. The median age at repair was 60 years, 108 (94%) had repair under general anaesthesia (GA), while only 7 (6%) were repaired using local infiltration anaesthesia (LA) and all were over 65 age groups. Modality of surgery varied; 72 (63%) were open procedures and 43 (37%) trans-abdominal pre-peritoneal (TAPP) laparoscopic repairs. Healthcare professional obtaining consent included; consultants 33 (29%), registrars 60 (52%), core trainees 20 (17%).

With regards to timing of written consent; 102 (89%) were recorded to have signed the consent form on the day of operation, 11 (10%) in the clinic, none in the pre-operative clinic. Pre-printed consent templates were used in 53 (46%), while 60 (60%) were hand-written. Only 16 (15%) were recorded to have received the information leaflet.

The response rate to the self-administered postal survey was 53% (n = 61) of completed questionnaires.

### 3.1 Audit

**Fig. 1:**
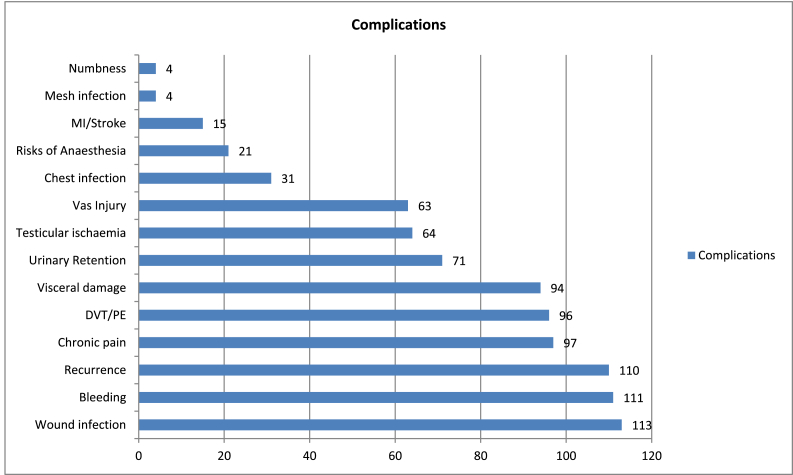
Overall documentation of risks in the consent forms (n = 113).

102 (89%) patients appeared to have signed and therefore completed the consent process on the day of operation and 11 (10%) in the outpatient clinic. In 29 (25%) cases, there was documented evidence of patient engagement in the decision making process. Documentation of overall information about the risks of operation in the consent form is shown in the Fig. 1. Pre-printed consent template-stickers as opposed to hand-written were used in 53 (46%) of cases (Table 2).

In over 75 (66%) cases, there was documentation of complete information about the risks, benefits and alternatives to surgery. In 7 (6%) cases no benefit was documented - each case used hand-written consent forms as opposed to template-stickers. In 104 (90%) cases, medical jargon (e.g. laparoscopic/open repair of inguinal hernia) was used for the description of the procedure rather than a “lay description” of surgery (e.g. repair of the weakness via a small cut or keyhole). Delegated consent was obtained by a junior doctor in 20 (18%) cases and subsequent of documentation of confirmation by the operating surgeon was unclear. Only 81 (70%) patients received information about the full benefits of surgery (to relieve symptoms and prevent complications), 27 (23%) patients received partial information (either relief of symptoms, prevent complications or repair the hernia) and in 7 (6%) no mention of benefit was recorded. Information about the immediate postoperative recovery was variably documented, with full detail provided to 85 (74%) patients, partial detail 17 (15%) and no documented advice to 13 (11%) patients.

All patients with documented recovery information featured consent forms using a pre-printed template sticker. 18 (16%) patients did not receive a carbon copy of the consent form to retain during the consent process; all of these patients had signed the consent form on the day of surgery.

### 3.2 Patient's survey

The results of patient questionnaire are shown in Table 3. The most common chosen preference for place and timing of consent was the clinic consultation with 39 (64%) responders, followed by the day of surgery 15 (25%), and no ranked preference 7 (11%). Perceived satisfaction was non-significantly lower in those signed the consent form on the day of surgery (76% versus 87%, p = 0.74). 49 (80%) felt happy with the overall consent process in terms of: sufficient time, no duress, and suitable explanations from the Surgeon. Only 34 (56%) of responders believed they experienced choice over modality of surgery, this was not statistically different whether the consent was done in the clinic or on the day of surgery.

**Table 3:** Patient questionnaire response.

Survey questionnaire	Day of operation	In clinic	P- value	Total
(Response: n = 61)	38 (62%)	23 (38%)		61 (100%)
Adequate time, no duress, could easily understand	29 (76%)	20 (87%)	0.507	49 (80%)
Patient felt choice of procedure given	20 (53%)	14 (61%)	0.601	34 (56%)
Anaesthesia explained in simple words	29 (76%)	22 (96%)	0.042	51 (84%)
Choice of anaesthesia given	12 (32%)	10 (43%)	0.414	22 (36%)
Pain control explained	25 (66%)	21 (91%)	0.032	46 (75%)
Recovery explained	22 (58%)	18 (78%)	0.164	40 (66%)
Support & advice received	35 (92%)	22 (96%)	1.000	57 (93%)
Leaflet received	32 (84%)	21 (91%)	0.698	53 (89%)
Felt confidence in NHS	37 (97%)	22 (96%)	1.000	59 (97%)
Prefer consent on the day of operation	15 (39%)	0 (0%)	1.000	15 (25%)
Prefer consent in the clinic	18 (47%)	21 (91%)	0.007	39 (64%)
Prefer consent anytime	5 (13%)	2 (13%)	0.700	7 (11%)
Confidence in NHS	37 (97%)	23 (100%)	1.000	60 (98%)
Would recommend to friends/family	36 (90%)	23 (100%)	0.522	59 (97%)

Overall 51 (84%) felt that the type of anaesthesia was explained, this was more likely if the consent was done in the clinic (96% versus 76%, p = 0.042). 53 (89%) confirmed that they were given an information leaflet by the Surgeon. 57 (93%) felt that they received support and advice. 60 (98%) responders felt confidence in the National Health Service and 59 (97%) would recommend treatment to family and friends.

## 4 Discussion

In clinical practice, consent for intermediate and minor procedures is often deferred to the day of surgery, commonly taken on a ward without due regard to privacy. Consent is sometimes delegated to junior medical staff that may not have sufficient knowledge of the procedure or the risks involved, with studies having shown that many consent procedures are incomplete [12,13]. Approximately 10% of litigation relates to lack of informed consent, with inguinal hernia repair being amongst the most common claims [14].

Data collection for our audit was retrospective; documentation of risk disclosure was assessed. In many cases, medical jargon was used for the procedure rather than a layman's description for the procedure. Majority of patients appeared to have signed the written consent form on the day of surgery and about one sixth were obtained by core trainees (delegated consent). We found differences in documentation of individual consenting practices and some variation in risk disclosure. This was largely due to variations in the documentation; hand written versus use of different consent templates in different sites. This causes variation in the information provided to the patients. Hand written information is usually incomplete, varies from surgeon to surgeon. Consent template ensures 100% documentation provided it has all the required information. In our consent templates mesh infection, numbness, testicular ischaemia resulting in testicular atrophy in general and port site hernia and possibility of contra-lateral repair during laparoscopic approach were not included. There was poor documentation of these risks being disclosed, these complications may have significant impact on patient's life and could potentially trigger negligence claims. Griffin et al. demonstrated in a series of 206 patients of clinically unilateral hernia, undergoing trans-abdominal pre-peritoneal (TAPP) approach that 45 (22%) had an occult contralateral hernia [15]. If the patient had not been consented for the possibility of contra lateral repair, repair of such occult hernia may be legally questionable and leaving incidental contra lateral hernia would exposed to the risks of a second operation, should this becomes symptomatic in future.

The use of pre-printed consent and discharge summary templates are superior with regards to the documentation of information given to the patients and improved the compliance with the best practice guidance. The study however, shows similar findings to earlier studies that the consenting practice for a common day case procedure like groin hernia repair needs improvement [13,14,16,17]. Although over 60% patients would prefer consent in the clinic and over half felt they were not actively involved in the choice of procedure or anaesthetic, suggesting a degree of medical paternalism, the satisfaction levels were higher (98%) whichever approach was used and over 97% patients would recommend treatment to friends and families.

Most of the patients undergoing groin hernia repair would have an uneventful recovery, but a small fraction will end up having complications ranging from self-limiting chronic pain to significant long-term morbidities. Generally, patient education, effective physician-patient communication and increased patient empowerment lead to improved outcomes [18,19]. Many patients do not retain verbal information effectively and a better quality of informed consent can be obtained by combining oral with written information [20,21]. When consent is sought prior to the day of operation, patient's recall of information and their satisfaction with the consent process is higher. It has been shown that the amount of time taken to consent patient improves patient's understanding, and decision making process [22].

Obtaining the signed written consent form is mandatory requirement by all the hospitals; it completes the consent process and provides some evidence of a contract between the patient and the doctor. Consenting process should be completed in the clinic. Seeking consent at the last minutes before surgery can be very distracting for both patient and the surgeon. Pressure of time on the surgeon can affect the quality of consent process. The patient may feel under duress or psychological commitment to proceed with an operation which he/she following the necessary disclosure would have either rejected or sought a second opinion.

Consent can be achieved by an initial consultation where the proposed procedure is discussed, the information leaflet is provided followed by a second discussion at the preoperative clinic and delegated consent obtained by either the nurse or junior doctor, who has had procedure specific consent training. Alternatively an information leaflet may be sent to the patient along with clinic appointment letter well in advance; such that the patient has full knowledge of the condition and treatment options to facilitate an informed decision process in the clinic consultation. We suggest, a standard pre-printed consent template, a separate discharge summary template for immediate postoperative care, particularly red flag signs, symptoms, wound care, and resumption of routine activities. These measures will improve the documentation of information and hence compliance with best practice but are not replacement for a full discussion of the various aspects of consent process.

Most of the studies on the quality of consenting process are reliant on the documentation in the medical notes which is historically inadequate. These only assess the process through doctor's perspective. Our study is unique that we have combined this with survey of the same patient cohort to investigate the patient's view of the process. This study confirms that use pre-printed consent sticker, discharge summary templates improves compliance with best practice guidance and completing consent process in the clinic before the day of surgery is in line with person centered approach.

The main limitations of this study include retrospective data collection, and reliance on documentation to determine the content of verbal consent process. For the survey, it was assumed that all the patients who were competent to sign the consent had understood, retained and weighed the information.

## Disclosures

Declaration of prior publication: It is to confirm that all Authors have seen and approved the manuscript being submitted. The article is the Authors' original work. The article has not received prior publication and is not under consideration for publication elsewhere. On behalf of all Co-Authors, the corresponding Author shall bear full responsibility for the submission. Authors declare that parts of this article are accepted as abstracts for oral presentation and e-Poster in the forthcoming conference of Association of Surgeons of Great Britain and Ireland being held on 9–11 May 2018.

## Human rights statements and informed consent

Local clinical audit standards and effectiveness board was informed prior to commencement, ethical approval was not required. For the survey; an invitation letter with full information was sent in the post, the response sheet was completed anonymously. The positive response from a respondent was considered in itself as evidence of consent.

## Ethical approval

Not required.

Local clinical audit standards and effectiveness board was informed prior to commencement, ethical approval was not required.

## Sources of funding

Self funded.

## Author contribution

Saad U. Khan (Corresponding author): study design, data collection, analysis and writing.

David J. Bowrey: study design, writing, proof reading.

Robert N. Williams: study design, writing, proof reading.

Jun Yi Soh: data analysis, writing.

Aikaterini Peleki: data collection, proof reading.

Nazli Muhibullah: data collection, data analysis, proof reading.

Peter W. Waterland: Data analysis, writing & proof reading.

## Conflicts of interest

All authors declare that they have no conflict of interest.

## Research registration number

Researchregistry 4379.

## Guarantor

**Saad U Khan** (Corresponding author).

This article is the Authors' original work. I have access to all the data and have full control over decision to publish. It is to confirm that all Authors have seen and approved the manuscript being submitted. The article has not received prior publication and is not under consideration for publication elsewhere. On behalf of all Co-Authors, as a corresponding Author, I shall bear full responsibility for the submission.

## Provenance and peer review

Not commissioned, externally peer-reviewed.
